# Cross-sectional and longitudinal study of association between circulating thiobarbituric acid-reacting substance levels and clinicobiochemical parameters in 1,178 middle-aged Japanese men - the Amagasaki Visceral Fat Study

**DOI:** 10.1186/1743-7075-8-82

**Published:** 2011-11-22

**Authors:** Yukiyoshi Okauchi, Ken Kishida, Tohru Funahashi, Midori Noguchi, Tomoko Ogawa, Kohei Okita, Hiromi Iwahashi, Tetsuya Ohira, Akihisa Imagawa, Tadashi Nakamura, Iichiro Shimomura

**Affiliations:** 1 Department of Metabolic Medicine, Graduate School of Medicine, Osaka University, Suita, Osaka 565-0871, Japan; 2 Department of Metabolism and Atherosclerosis, Graduate School of Medicine, Osaka University, Suita, Osaka 565-0871, Japan; 3 Amagasaki City Office, General Affairs Bureau, Personal Department, Payroll Section, Employee Health Promotion Section, Amagasaki, Hyogo 660-8501, Japan; 4 Department of Social and Environmental Medicine, Graduate School of Medicine, Osaka University, Suita, Osaka 565-0871, Japan

**Keywords:** visceral fat accumulation, systemic reactive oxidative stress, visceral fat reduction

## Abstract

**Background:**

Circulating thiobarbituric acid-reacting substance (TBARS) levels, a marker of systemic oxidative stress, are predictive of cardiovascular events. However, they has not been evaluated in Japanese, especially with regard to the factors that contribute to the changes in circulating TBARS levels. We investigated the cross-sectional and longitudinal relationships between circulating TBARS levels and various clinicobiochemical parameters in middle-aged men.

**Methods:**

In this population-based study (The Amagasaki Visceral Fat Study), 1,178 Japanese male urban workers who had undergone health check-ups in 2006, 2007 and 2008 and were not on medications for metabolic disorders during the follow-up period, were enrolled. Serum TBARS levels were measured by the method of Yagi. The estimated visceral fat area (eVFA) by bioelectrical impedance was measured annually. After health check-ups, subjects received health education with lifestyle modification by medical personnel.

**Results:**

The number of cardiovascular risk factors (hypertension, hyperglycemia, low HDL-C, hypertriglyceridemia, hyperuricemia, hyper-LDL-C and impaired renal function) augmented with the increases in log-eVFA (p < 0.0001) and log-TBARS (p < 0.0001). The combination of TBARS and eVFA had a multiplicative effect on risk factor accumulation (F value = 79.1, p = 0.0065). Stepwise multiple regression analysis identified log-eVFA, as well as age, log-body mass index (BMI), LDL-C, log-adiponectin, γ-glutamyl transpeptidase (γ-GTP) and uric acid as significant determinants of log-TBARS. Stepwise multiple regression analysis identified one-year changes in eVFA as well as BMI, γ-GTP and estimated glomerular filtration rate (eGFR) as significant determinants of one-year change in TBARS, and biennial changes in eVFA as well as BMI and γ-GTP, eGFR as significant determinants of biennial change in TBARS.

**Conclusions:**

The present study showed a significant cross-sectional and longitudinal correlation between TBARS and eVFA, as well as BMI and γ- GTP, eGFR. Visceral fat reduction may independently associate with the improvement in systemic ROS in middle-aged Japanese men.

**Trial Registration:**

The Amagasaki Visceral Fat Study UMIN000002391.

## Background

Oxidative stress results from an imbalance between reactive oxygen species (ROS) and antioxidants, and is influenced by genetic and environmental factors. In the general population, serum levels of thiobarbituric acid-reacting substance (TBARS), an important biomarker of systemic oxidative stress, were reported to be associated with various factors, such as age, body mass index (BMI), glucose metabolism, lipid metabolism [1], γ-glutamyl transpeptidas (γ-GTP) [2], uric acid (UA) [3] and smoking, and the levels correlated with atherosclerogenesis [4]. Serum levels of TBARS were strongly predictive of cardiovascular events in patients with stable coronary artery disease, independently of traditional risk factors [5,6].

We reported previously that adipose tissue is the major source of ROS (called FatROS), and that systemic oxidative stress is closely associated with fat accumulation in obese animals [7]. These animals also exhibited selective overproduction of ROS in adipose tissue, as well as over-expression of NADPH oxidase and under-expression of antioxidative enzymes [7]. We and others have demonstrated that increased FatROS and systemic oxidative stress are the underlying causes of dysregulated production of adipocytokines (e.g., hypoadiponectinemia and high levels of plasminogen activator inhibitor-1, interleukin-6, and monocyte chemoattractant protein-1), which might lead to the development of lifestyle-related diseases [7,8]. In obese mice, treatment with NADPH oxidase inhibitors reduced ROS production by the adipose tissue, attenuated the dysregulation of adipocytokines, and improved diabetes and hyperlipidemia [7]. However, the level of systemic ROS production has not been evaluated properly in the general population, especially with regard to the factors that contribute to the changes in systemic ROS production.

The present cross-sectional and longitudinal study investigated the relationship between circulating TBARS levels and various clinicobiochemical parameters in middle-aged Japanese male urban workers.

## Methods and Procedures

### Participants

The study subjects were 1,178 Japanese men [age; mean ± SD 45 ± 10 (range, 20-68) years], who were employees of the Amagasaki city office and had undergone annual health check-ups in three consecutive years (2006, 2007 and 2008). To avoid the influence of medications, we excluded subjects who were on treatment for diabetes, hypertension and dyslipidemia during the 2-year follow-up period. Table 1 left summarizes the profiles of all participants at baseline. After the health check-up, the medical staff, especially health nurses, provided the individual with annual health promotion program to promote voluntary lifestyle changes (2006 and 2007) [9]. In brief, the program emphasized hazards of visceral fat accumulation and multiple cardiovascular risk factors, with the aim of encouraging a scientific understanding of the concept of metabolic syndrome from visceral fat accumulation to the development of atherosclerotic cardiovascular diseases. We reported previously that this program should be useful in reducing visceral fat accumulation and consequently decrease number of cardiovascular risks, such as glucose tolerance, dyslipidemia, high blood pressure [10], hypoadiponectinemia [11], and a prevalence of the metabolic syndrome [9], leading to prevention of cardiovascular events [12,13].

**Table 1:** Baseline characteristics of the studied population (n = 1,178)

	year 2006 (n = 1,178)		year 2007 (n = 1,178)		year 2008 (n = 1,178)	
Age (year)	45 ± 10	(20-68)	46 ± 10	(21-69)	47 ± 10	(22-70)
Body weight (kg)	68.8 ± 9.5	(43.7-118.5)	68.8 ± 9.6	(44.4-124.5)	68.7 ± 9.7	(44.0-118.1)
Body mass index (kg/m^2^)	23.8 ± 2.9	(16.4-37.6)	23.8 ± 3.0	(15.9-39.4)	23.8 ± 3.0	(16.0-37.1)
Waist circumference (cm)	82.5 ± 8.1	(59-119)	82.5 ± 8.2	(60-117)	81.9 ± 8.0	(59-119)
Estimated visceral fat area (cm^2^)	88.5 ± 38.5	(14-304)	89.8 ± 39.3	(4-290)	87.8 ± 37.8	(4-316)
Systolic blood pressure (mmHg)	123 ± 14	(86-174)	122 ± 15	(90-186)	122 ± 16	(87-190)
Diastolic blood pressure (mmHg)	75 ± 10	(45-120)	75 ± 11	(46-110)	75 ± 11	(51-116)
Total cholesterol (mmol/L)	5.17 ± 0.85	(2.6-8.7)	5.20 ± 0.86	(2.8-8.5)	5.19 ± 0.85	(2.5-8.6)
Triglyceride (mmol/L)	1.61 ± 1.36	(0.25-23.8)	1.65 ± 1.21	(0.24-10.6)	1.62 ± 1.25	(0.24-16.8)
High-density lipoprotein-cholesterol (mmol/L)	1.43 ± 0.34	(0.62-2.75)	1.50 ± 0.38	(0.57-3.39)	1.50 ± 0.39	(0.39-2.93)
Low-density lipoprotein-cholesterol (mmol/L)	3.00 ± 0.75	(0.93-6.14)	3.23 ± 0.80	(0.91-6.68)	3.12 ± 0.78	(0.67-5.88)
Hemoglobin A1c (%), JDS	5.1 ± 0.6	(3.0-10.8)	5.1 ± 0.5	(3.5-10.2)	5.2 ± 0.5	(3.3-9.9)
γ-GTP (μkat/L)	0.48 ± 0.55	(0.10-6.30)	0.47 ± 0.57	(0.08-8.85)	0.49 ± 0.63	(0.09-6.81)
Creatinine (μmol/L)	77.8 ± 9.8	(49.5-116.7)	75.8 ± 10.1	(43.3-113.2)	75.4 ± 10.0	(48.6-116.7)
eGFR (mL/min/1.73 m^2^)	77.0 ± 11.8	(44.6-124.9)	78.8 ± 12.5	(46.6-135.5)	78.8 ± 12.6	(47.7-138.0)
Uric acid (mmol/L)	362.0 ± 71.8	(136.8-618.6)	361.0 ± 72.6	(124.9-672.2)	360.5 ± 70.3	(124.9-636.5)
TBARS (nmol/mL)	7.6 ± 2.3	(3.0-20.1)	7.7 ± 2.0	(3.7-25.3)	5.9 ± 1.6	(2.8-18.7)
Adiponectin (μg/mL)	6.7 ± 3.1	(0.8-31.8)	7.0 ± 3.3	(0.7-37.3)	7.3 ± 3.7	(0.6-39.1)
Smoking						
Current-smoker	n = 460	39.0%	n = 442	37.5%	n = 366	31.1%
Ex-smoker	n = 172	14.6%	n = 186	15.8%	n = 170	14.4%
Non-smoker	n = 546	46.4%	n = 550	46.7%	n = 642	54.5%

Data are mean ± SD (range) or numbers (n) of subjects.

γ-glutamyl transpeptidase (γ-GTP), eGFR; estimated glomerular filtration rate, TBARS; thiobarbituric acid reactive substances.

### Anthropometry and laboratory measurements

Body weight (kg), height (cm), and waist circumference at umbilical level (cm) were measured in standing position, and systolic and diastolic blood pressures (SBP, DBP, respectively) were measured with a standard mercury sphygmomanometer after rest in sitting position for at least 5 minutes. BMI was calculated using the formula [weight (kg)/height (m) ^2^]. Visceral fat area was estimated by bioelectrical impedance analysis (eVFA), as we reported previously [14]. After overnight fasting or at least 5 hours-fasting, venous blood samples were collected for measurements of blood glucose (BS), hemoglobin A1c (HbA1c, National Glycohemoglobin Standardization Program), triglyceride (TG), high-density lipoprotein-cholesterol (HDL-C), high-density lipoprotein-cholesterol (LDL-C), γ-glutamyl transpeptidase (γ-GTP), creatinine, uric acid (UA) and adiponectin (Otsuka Pharmaceutical Co., Tokushima, Japan), while the subject was in sitting position. For the purpose of the present study, serum samples that were obtained at baseline from each participant and stored promptly at -20°C without the addition of exogenous antioxidants before TBARS assay. After thawing the samples, serum levels of malondialdehyde in terms of TBARS, a marker of systemic ROS production, were measured in duplicate in each of 3,534 samples by the method of Yagi (Japan Institute for the Control of Aging, Nikken SEIL Co., Shizuoka, Japan) [15], as we [16,17] and other [18] previously reported. The selection of this parameter was based on the fact that malondialdehyde can be generated from oxidative mechanisms other than lipid peroxidation, and that TBARS is assumed to represent a composite of systemic oxidative damage products, including malondialdehyde [18]. Glomerular filtration rate was estimated by the following equation (eGFR = 194 × serum creatinine ^-1.094 ^× age ^-0.287^).

We investigated the presence of seven cardiovascular risk factors: 1) hypertension (SBP ≥ 130 and/or DBP ≥ 80 mmHg) [19], 2) hyperglycemia (fasting or postprandial BS of ≥ 6.10 or 7.77 mmol/L, respectively) [19], 3) low HDL-C (HDL-C < 1.04 mmol/L) [19], 4) hypertriglyceridemia (fasting or postprandial TG of ≥ 1.69 or 2.27 mmol/L, respectively) [19], 5) hyperuricemia (UA ≥ 416 μmol/L), 6) hyper-LDL-cholesterolemia (LDL-C ≥ 3.64 mmol/L) and 7) impaired renal function (eGFR < 60 mL/min/1.73 m^2^).

### Statistical analysis

**Figure 1:**
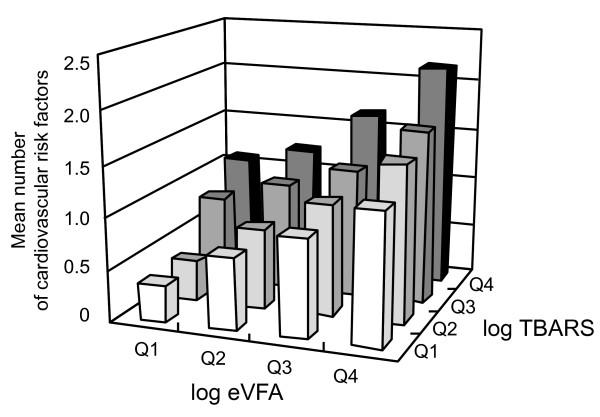
**Mean number of cardiovascular risk factors in the study population in relation to the log estimated visceral fat area (log-eVFA) and log-circulating thiobarbituric acid-reacting substance levels (log-TBARS)**. Each parameter was categorized into quartiles (Q1-Q4).

**Figure 2:**
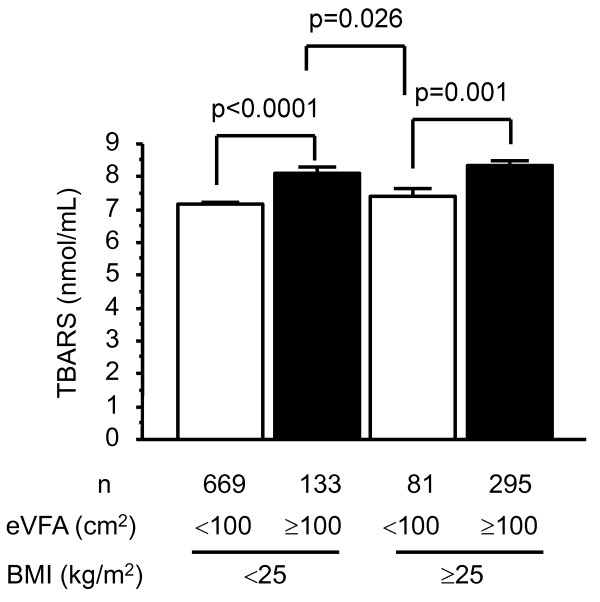
**Relationship between circulating TBARS levels and body fat distribution**. Subjects were divided according to body mass index (BMI) using a cutoff value of 25 kg/m^2 ^and estimated visceral fat area (eVFA) using a cutoff value of 100 cm^2^, measured in 2006. Data are mean ± SEM. TBARS: thiobarbituric acid-reacting substance.

Data of TBARS, eVFA and adiponectin levels showed skewed distribution, and were thus log-transformed before analysis. Pearson's correlation coefficient was used to examine the relationship between log-TBARS and various clinicometabolic parameters, and between one-year and biennial changes in TBARS and various clinicometabolic parameters. Significant level was set at p < 0.05. Stepwise multiple regression analysis was conducted to identify the parameters that significantly contributed to log-TBARS or one-year and biennial changes in TBARS. Parameters with F value > 4.0 were subsequently entered into regression analysis as independent variables. Differences in the mean number of obesity-related cardiovascular risk factors between eVFA and TBARS were analyzed by the Kruskal-Wallis test (Figure 1). Differences among groups were compared by unpaired Student's t-test for experiments involving only two groups (Figure 2). Continuous variables were expressed as mean ± SEM (Figure 2) or SD (Table 1). All statistical analyses were performed with the Statistical Package for Social Sciences (vesion 11.0.1J; SPSS, Chicago, IL).

### Ethics Approval

The study was approved by the human ethics committee of Osaka University and a signed informed consent was obtained from each participant, based on the ethical guideline of the 2000 Declaration of Helsinki of the World Medical Association.

## Results

### The cross-sectional study

First, we compared the mean number of cardiovascular risk factors (hypertension, hyperglycemia, low HDL-C, hypertriglyceridemia, hyperuricemia, hyper-LDL-C and impaired renal function) after classifying the subjects according to the log-eVFA and log-TBARS values. The number of risk factors increased significantly with the increase in eVFA (p < 0.0001 for trend, the Kruskal-Wallis test). Moreover, the number of cardiovascular risk factors increased significantly with increased levels of circulating TBARS (p < 0.0001 for trend, the Kruskal-Wallis test) (Figure 1). We investigated the supra-additive effect of TBARS and eVFA on cardiovascular risk factor accumulation using age-adjusted multiplicative interaction term (log TBARS × log eVFA) in the linear regression model, and log TBARS and log-eVFA individually. Multiple linear regression analysis (adopted factors; age, log TBARS, log-eVFA, log TBARS × log eVFA) identified interaction term (log TBARS × log eVFA) as a significant determinant of cardiovascular risk factor accumulation (F value = 79.1, p = 0.0065). These results indicated that the combination of TBARS and eVFA had a multiplicative effect on cardiovascular risk factor accumulation (Figure 1).

Second, analysis of data collected in year 2006 showed positive relationships between log-TBARS and the following parameters: age, BMI, waist circumference, log-eVFA, SBP, DBP, LDL-C, HbA1c, γ-GTP, ex-/current-smoking and UA, and a negative relationship between log-TBARS and log-adiponectin and eGFR (Table 2). Stepwise multiple regression analysis identified age, LDL-C, log adiponectin, γ-GTP, UA and log-eVFA [multivariate 1 (eVFA); adopted factors: age, eVFA SBP, LDL-C, HbA1c, log-adiponectin, γ-GTP, eGFR, UA, smoking status] or log-BMI [multivariate 2 (BMI); adopted factors; age, BMI SBP, LDL-C, HbA1c, log-adiponectin, γ-GTP, eGFR, UA, smoking status], as significant determinants of log-TBARS (Table 2).

**Table 2:** Cross-sectional results of correlation between log-TBARS and various parameters by uni- and multi-variate analyses

Log-TBARS	Univariate		Multivariate1 (eVFA)			Multivariate2 (BMI)		
	**r**	**p value**	**F value**	**r**	**p value**	**F value**	**r**	**p value**

Age	0.282	< 0.0001	55.452	0.203	< 0.0001	76.055	0.231	< 0.0001
BMI	0.211	< 0.0001	-	-	-	9.681	0.086	0.0012
WC	0.257	< 0.0001	-	-	-	-	-	-
Log-eVFA	0.307	< 0.0001	15.023	0.115	0.0001	-	-	-
SBP	0.147	< 0.0001	0.066	-	-	0.026	-	-
DBP	0.212	< 0.0001	-	-	-	-	-	-
HDL-C	-0.050	0.0855	-	-	-			
LDL-C	0.262	< 0.0001	45.483	0.184	< 0.0001	48.025	0.189	< 0.0001
HbA1c	0.156	< 0.0001	1.734	-	-	1.587	-	-
Log-adiponectin	-0.190	< 0.0001	10.317	-0.089	0.0014	13.885	-0.101	0.0022
γ-GTP	0.253	< 0.0001	34.293	0.163	< 0.0001	37.444	0.170	< 0.0001
eGFR	-0.149	< 0.0001	0.078	-	-	0.000	-	-
UA	0.213	< 0.0001	16.529	0.112	< 0.0001	16.493	0.120	< 0.0001
Smoking (Current-)	-	0.1235						
Smoking (Ex- and Current-)	-	0.0159	0.0002	-	-	0.011	-	-

Univariate: Pearson's correlation analysis, Multivariate: Stepwise multiple regression analysis.

Parameters with F value > 4.0 were subsequently entered into the regression analysis as independent variables.

BW: Body weight, BMI: body mass index, WC: waist circumference, Log-eVFA: estimated visceral fat area,

SBP: systolic blood pressure, DBP: diastolic blood pressure, HDL-C: high-density lipoprotein-cholesterol,

LDL-C: low-density lipoprotein-cholesterol, HbA1c: Hemoglobin A1c, eGFR: estimated glomerular filtration rate,

UA: uric acid.

Next, to clarify whether the degree of obesity or body fat distribution relates more strongly with circulating TBARS levels, we divided subjects into four groups according to BMI (cutoff value 25 kg/m^2^) and eVFA (cutoff value 100 cm^2^), based on Japanese criteria of obesity and visceral fat accumulation [20] (Figure 2). Subjects with eVFA ≥ 100 cm^2 ^had significantly higher levels of circulating TBARS than those with eVFA <100 cm^2^, irrespective of BMI (p < 0.0001, respectively). Interestingly, subjects with visceral fat accumulation but without overall obesity (eVFA ≥ 100 cm^2 ^plus BMI < 25 kg/m^2^) had significantly higher levels of circulating TBARS than those without visceral fat accumulation but with overall obesity (eVFA <100 cm^2 ^plus BMI ≥ 25 kg/m^2^) (p = 0.026). These results indicate that body fat distribution, rather than with the degree of obesity, should be more associated with circulating TBARS levels.

### The longitudinal study

We also investigated the relationship between one-year changes in TBARS (one-year ΔTBARS) and various parameters (Table 3A). The one-year ΔTBARS correlated positively with one-year ΔBMI, ΔeVFA, Δγ-GTP, ΔeGFR, and ΔUA. Stepwise multiple regression analysis identified one-year Δγ-GTP, ΔeGFR, log-eVFA [multivariate 1 (one-year ΔeVFA); adopted factors; one-year ΔeVFA, Δγ-GTP, ΔeGFR, ΔUA] and log-BMI [multivariate 2 (one-year ΔBMI); adopted factors; one-year ΔBMI, Δγ-GTP, ΔeGFR, ΔUA] as significant determinants of one-year ΔTBARS.

**Table 3:** Longitudinal results of uni- and multi-variate analyses

Correlation between one-year ΔTBARS and Δvalues of various parameters								
**One-year Δ TBARS**	**Univariate**		**Multivariate1 (eVFA)**			**Multivariate2 (BMI)**		

	**R**	**p value**	**F value**	**r**	**p value**	**F value**	**r**	**p value**

Δ BMI	0.105	0.0003	-	-	-	10.389	0.092	0.0013
Δ WC	0.054	0.0634	-	-	-	-	-	-
Δ eVFA	0.090	0.0020	5.278	0.066	0.0218	-	-	-
Δ SBP	-0.042	0.1480						
Δ DBP	0.025	0.3822						
Δ HDL-C	-0.006	0.8320						
Δ LDL-C	0.047	0.1099						
Δ HbA1c	0.039	0.1864						
Δ adiponectin	-0.018	0.5348						
Δ γ-GTP	-0.132	< 0.0001	17.775	-0.121	< 0.0001	17.994	-0.121	< 0.0001
Δ eGFR	-0.154	< 0.0001	27.577	-0.150	< 0.0001	28.731	-0.153	< 0.0001
Δ UA	0.086	0.0033	0.001	-	-	0.013	-	-

**Correlation between biennial ΔTBARS and Δvalues of various parameters**								

**Biennial Δ TBARS**	**Univariate**		**Multivariate1 (eVFA)**			**Multivariate2 (BMI)**		

	**r**	**p value**	**F value**	**r**	**p value**	**F value**	**r**	**p value**

Δ BMI	0.109	0.0002	-	-	-	10.243	0.093	0.0014
Δ WC	0.091	0.0018	-	-	-	-	-	-
Δ eVFA	0.094	0.0013	6.433	0.074	0.0113	-	-	-
Δ SBP	-0.040	0.1732						
Δ DBP	0.003	0.9147						
Δ HDL-C	-0.045	0.1222						
Δ LDL-C	0.078	0.0077	3.759	-	-	3.073	-	-
Δ HbA1c	0.008	0.7720						
Δ adiponectin	-0.020	0.4970						
Δ γ-GTP	-0.103	0.0004	9.627	-0.090	0.0020	9.282	-0.088	0.0024
Δ eGFR	-0.115	< 0.0001	14.417	-0.109	0.0002	14.835	-0.111	0.0001
Δ UA	0.084	0.0037	0.285	-	-	0.255	-	-

Moreover, we also investigated the relationships between biennial changes in TBARS (biennial ΔTBARS) and various parameters (Table 3B). Biennial ΔTBARS correlated with biennial ΔBMI, Δwaist circumference, ΔeVFA, ΔLDL-C, Δγ-GTP, ΔeGFR, and ΔUA. Stepwise multiple regression analysis identified biennial Δγ-GTP, ΔeGFR, log-eVFA [multivariate 1 (biennial ΔeVFA); adopted factors; biennial ΔeVFA, ΔLDL-C, Δγ-GTP, ΔeGFR, ΔUA] and log-BMI [multivariate 2 (biennial DBMI); adopted factors; biennial ΔBMI, ΔLDL-C, Δγ-GTP, ΔeGFR, ΔUA], as significant determinants of biennial ΔTBARS (Table 3B).

## Discussion

The cross-sectional study is the first to report that body fat distribution, i.e. visceral fat accumulation, as well as age, BMI, LDL-C, γ-GTP and UA as reported previously [1-3], is important determinant of circulating TBARS levels in Japanese men (Table 2). Moreover, subjects with visceral fat accumulation had significantly higher levels of circulating TBARS than those without visceral fat accumulation, both in those with and without overall obesity (Figure 2), as previous report demonstrated that visceral fat thickness and serum TBARS levels [21]. The results also emphasize the importance of visceral adipose tissue *per se *as the major source of ROS in the whole body in the general population, as we reported previously in obese subjects [7] and in subjects at high risk for cardiovascular diseases [22]. This present study demonstrated that visceral fat may be the major source of systemic oxidative stress, and that visceral fat accumulation increased systemic oxidative stress with the underlying causes of cardiovascular risk factor accumulation (Figure 1) and dysregulated production of adipocytokines (e.g., hypoadiponectinemia) (Table 2), which might lead to the development of lifestyle-related disease including coronary artery disease.

Another aim of the present study was to clarify whether reduction of visceral fat associated with reductions in systemic oxidative stress. We reported previously that visceral fat reduction through lifestyle modification reduced the number of atherosclerotic cardiovascular events [12]. The present longitudinal study indicated that weight loss and reduction of visceral fat *per se *through lifestyle modification was associated with reductions in systemic oxidative stress (Table 3) accompanied by improvement in cardiovascular risk accumulation, which probably lessens the risk of atherosclerotic cardiovascular diseases. Life-style modification designed to effectively reduce the amount of visceral fat, which is associated with improvement in liver and renal dysfunction, is probably beneficial in reducing systemic ROS overload, leading to combat cardiovascular disease events in human. There is no doubt that other factors also influence the status of systemic oxidative stress, such as physical activity, alcohol consumption, socio-economic status, sleep status, psychogenic stress status and dietary habits. These lifestyle changes themselves may also reduce circulating TBARS levels, although these effects were not analyzed in the present study.

The present study also showed a significant cross-sectional and longitudinal correlation between TBARS and markers of both liver and renal functions; γ-GTP and eGFR, respectively. γ-GTP is the enzyme responsible for extracellular catabolism of glutathione [2], and it has been used as a marker of excessive alcohol intake or of hepatic diseases [2]. γ-GTP, as a scavenger of oxygen free radicals, plays a key role in rather protecting against both intracellular and extracellular oxidative stress [23]. However, many epidemiological studies have suggested that increased γ-GTP levels may be a precocious and sensitive marker of oxidative stress, identifying persons with low, but persistent, augmentation of nonalcoholic fatty liver disease [24]. Serum TBARS levels are elevated in chronic kidney disease patients [25,26]. TBARS interferes with nitric oxide generation through competitive inhibition of nitric oxide (NO)-synthase enzyme, and therefore may lead to impairent in endothelium NO pathway in kidney [25-27]. Endothelial dysfunction is a common event described both in chronic and acute renal failure [27]. A possible direct association between TBARS and γ-GTP or eGFR should be examined by further experimental studies.

Our study has several limitations. First, the results may not be applicable to females or non-Japanese populations. Second, blood samples were collected at random rather than at fixed daytime. Multiple measurements on a like-for-like basis should be preformed for better assessment of TBARS. Third, during the 2-year follow-up (from 2006 to 2008), there was only one first-ever cardiovascular disease event. Monitoring the long-term effects of visceral fat reduction with lifestyle modification on the cumulative incidence of cardiovascular events is required. It is required to clarify the effects of the health promotion program on compliance and response and their potential influence on circulating TBARS. Further controlled studies of blind and randomized design should be also required. Fourth, smoking status was collected through a self-questionnaireis (non-, ex- or current-) without Brinkman index (daily number of cigarettes × years). Therefore, the present study could not investigate longitudinal study for smoking with reliability. Fifth, the current study did not include the effects of other important determinants of oxidative status, such as physical activity, alcohol consumption, smoking habits, socio-economic status, marital status, sleep status, dietary habit, and intake of antioxidants, such as ascorbic acid (vitamin C) and α-tocopherol (vitamine E), polyphenol and resveratrol (a red wine constituent) and pharmacological agents. Finally, the measurement of TBARS using the method of Yagi [14], particularly without an HPLC/GCMS clean-up to isolate the specific adduct, is limiting. This assay has limited sensitivity and specificity because TBA reacts with a variety of compounds (sugars, amino acids, bilirubin and albumin). More specific markers need to be measured [28].

In conclusion, the absolute value and sequential changes in systemic oxidative stress correlated significantly with those of adiposity, especially the amount of visceral fat, γ-GTP, and eGFR, in middle-aged Japanese men.

## List of Abbreviations

DBP: diastolic blood pressure; eGFR: estimated glomerular filtration rate; eVFA: estimated visceral fat area; γ-GTP: γ-glutamyl transpeptidase; HbA1c: hemoglobin A1c; HDL-C: high-density lipoprotein-cholesterol; LDL-C: low-density lipoprotein-cholesterol; ROS: reactive oxygen species; SBP: systolic blood pressure; TBARS: thiobarbituric acid-reacting substances; UA: uric acid

## Competing interests

The authors declare that they have no competing interests.

## Authors' contributions

YO and KK conducted the research, analyzed data, and wrote the manuscript. KK reviewed/edited the manuscript. MN and TO conducted the research. K, HI AI and TN contributed to the discussion. TO provided advice on statistical analysis. TF and IS contributed to the discussion and wrote the manuscript. All authors read and approved the final version of the manuscript.
